# Learning Abilities and Disabilities: Generalist Genes, Specialist Environments

**DOI:** 10.1111/j.1467-8721.2007.00521.x

**Published:** 2007-10

**Authors:** Yulia Kovas, Robert Plomin

**Affiliations:** Social, Genetic, and Developmental Psychiatry Centre, Institute of Psychiatry, King's College LondonLondon, England

**Keywords:** disabilities, reading, mathematics, genetics, multivariate

## Abstract

Twin studies comparing identical and fraternal twins consistently show substantial genetic influence on individual differences in learning abilities such as reading and mathematics, as well as in other cognitive abilities such as spatial ability and memory. Multivariate genetic research has shown that the same set of genes is largely responsible for genetic influence on these diverse cognitive areas. We call these “generalist genes.” What differentiates these abilities is largely the environment, especially nonshared environments that make children growing up in the same family different from one another. These multivariate genetic findings of generalist genes and specialist environments have far-reaching implications for diagnosis and treatment of learning disabilities and for understanding the brain mechanisms that mediate these effects.

Why do children differ in their ability to read, to use language, or to understand mathematics? One way to answer this question is to use genetic research methods to investigate genetic and environmental causes of such differences among children. Two decades of research make it clear that genetics is a surprisingly large part of the answer for both learning abilities and learning disabilities.

A review of twin studies of language disability reported concordance (the likelihood that one twin will be affected if the other twin is affected) of 75% for monozygotic (MZ, identical) twins and 43% for dizygotic (DZ, fraternal) twins (Stromswold, 2001). For reading disability, the concordances for MZ and DZ twins are 84% and 48%, respectively. For mathematics disability, the concordances are about 70% for MZ twins and 50% for DZ twins (Oliver et al., 2004). Such studies consistently indicate substantial heritability for learning abilities as well as for disabilities.

Genetic research has moved beyond merely demonstrating the importance of genetic influence to ask more interesting questions. Multivariate genetic analysis makes it possible to ask questions about the genetic and environmental links between and within learning abilities and disabilities. The analysis focuses on the covariance (correlation) between two traits (bivariate) or multiple traits (multivariate) and uses the twin method to estimate genetic and environmental contributions to their covariance as well as the variance of each trait. In other words, multivariate genetic analysis estimates the extent to which genetic and environmental factors that affect one trait also affect another trait. Although space does not permit a detailed explanation, Figure 1 illustrates the model used in multivariate genetic analyses. Such analyses yield the *genetic correlation*, a statistic central to this article. The genetic correlation (which may range from 0, no correlation, to 1.0) indexes the extent to which genetic effects on one trait correlate with genetic effects on another trait independently of the heritability of the two traits. As shown in Figure 1, multivariate genetic analyses also yield analogous shared and nonshared environmental parameters. Multivariate genetic research has produced surprising findings with far-reaching implications. The purpose of this article is to review the results and to consider those implications.

**Fig. 1:**
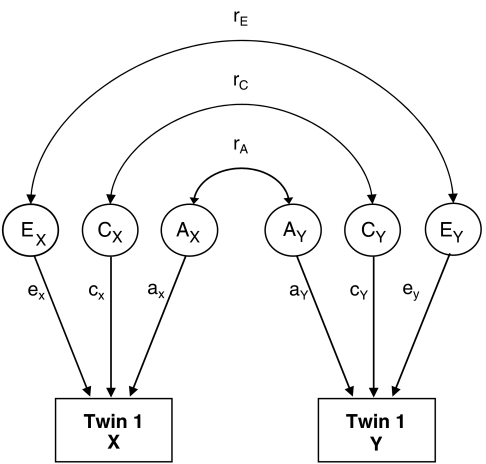
Correlated factors model for individual differences on traits X and Y in one individual from a twin pair. Though not illustrated here, there are genetic and shared environmental correlations between the two members of a pair for both X and Y scores. Using the twin method that compares monozygotic (MZ, identical) and dizygotic (DZ, fraternal) twin resemblance, variance in each trait is divided into that due to latent additive genetic influences (A), shared environmental influences (C), and nonshared environmental influences (E), with the subscripts x and y to denote scores on traits X and Y, respectively. Paths, represented by lower case (a, c, and e), are standardized regression coefficients and are squared to estimate the proportion of variance accounted for. The gist of the multivariate genetic method lies in cross-trait twin correlations. Just as univariate genetic analysis compares MZ and DZ correlations for a single trait, multivariate genetic analysis compares MZ and DZ correlations across traits. If MZ cross-trait cross-twin (CTCT) correlations are greater than DZ CTCT correlations, this suggests that genetic differences account for some of the phenotypic correlation between the traits. Correlations between the latent genetic, shared environmental, and nonshared environmental influences are denoted by r_A_, r_C_ and r_E_. The genetic correlation represents the extent to which genetic influences on trait X are correlated with genetic influences on trait Y regardless of the heritabilities of traits X and Y. Bivariate heritability, which represents the genetic contribution to the phenotypic correlation between traits X and Y, is the product of the paths a_x_r_a_a_y_, which weights the genetic correlation by the heritabilities of the traits.

## GENERALIST GENES FOR READING, MATHEMATICS AND LANGUAGE

Multivariate genetic research on learning abilities and disabilities consistently yields high genetic correlations. In a recent review, genetic correlations varied from .67 to 1.0 for reading versus language (five studies), .47 to .98 for reading versus mathematics (three studies) and .59 to .98 for language versus mathematics (two studies; Plomin & Kovas, 2005).

These studies examined the entire distribution of individual differences in learning abilities. What about disabilities? Few multivariate genetic studies of disabilities have been reported because they require large samples of twins for both types of disabilities in order to investigate their co-occurrence. In general, genetic research comparing abilities and disabilities suggests that what we call learning disability is merely the low end of the normal distribution of learning ability and caused by the same genetic and environmental factors responsible for learning ability (Plomin & Kovas, 2005). The implication is that when large multivariate genetic studies of disabilities are conducted, they will yield similarly high genetic correlations.

These high genetic correlations indicate that the genes affecting one ability (e.g., reading) are to a surprising extent the same genes that affect other abilities (e.g., mathematics). In order to highlight this general effect of genes, we refer to them as generalist genes. When DNA research identifies genes responsible for genetic influence on reading ability and disability, for example, we predict that most of these genes will also be associated with mathematics ability and disability because the genetic correlation between reading and mathematics is .70.

If genetic correlations are so high between learning abilities, it makes sense to expect that components within each learning domain are also highly correlated genetically, and that is the case. Genetic correlations range between .60 and .90 within each of the domains of language, reading, and mathematics (Plomin & Kovas, 2005). The most recent study used Web-based testing to assess five components of mathematics, including computation, interpretation, and non-numerical processes, in a study of more than 1,000 10-year-old twin pairs (Kovas, Petrill, & Plomin, 2007). The average genetic correlation between the five components of mathematics was .91.

It is important to emphasise that conclusions regarding generalist genes apply to common abilities and disabilities whose origins involve multiple genes and multiple environmental influences, not to rare single-gene disorders such as Phenylketonuria or chromosomal disorders such as Down syndrome. Furthermore, the generalist-genes hypothesis does not extend to rare or family-specific mutations, such as the FOXP mutation in the KE family, a family with an unusual type of speech-language impairment that includes deficits in oro-facial motor control. (Lai, Fisher, Hurst, Vargha-Khadem, & Monaco, 2001). The FOXP mutation appears to be both necessary and sufficient to cause this impairment in the 15 affected KE family members but does not contribute to genetic variation in common language disabilities (Meaburn, Dale, Craig, & Plomin, 2002).

Instead of thinking about rare genetic disorders caused by a single-gene mutation of the sort that Mendel investigated in the pea plant, it is now generally accepted that common disorders are caused by many genes, which implies that each of these genes will have only a small effect. These multiple genetic variants of small effect are called *quantitative trait loci* (QTLs), referring to the loci in the DNA that contribute to the variation in continuously (quantitatively) distributed traits. If, as is now generally accepted, disorders represent the quantitative extremes of the normal variation in complex traits, QTLs contribute to disorders interchangeably and additively as probabilistic risk factors.

## GENERALIST GENES FOR OTHER COGNITIVE ABILITIES

Much multivariate genetic research has focused on cognitive abilities, such as verbal, spatial, and memory abilities, rather than on learning abilities. This research consistently finds genetic correlations greater than .50 and often near 1.0 across diverse cognitive abilities (Deary, Spinath, & Bates, 2006). Similar results suggesting substantial genetic overlap have been found for more basic information-processing measures, such as speed of processing, as well as measures of brain volume (Deary et al., 2006).

Phenotypic (observed) correlations among diverse tests of cognitive abilities led Charles Spearman in 1904 to call this general factor *g* in order to avoid the many connotations of the word intelligence. To what extent do generalist genes for *g* overlap with generalist genes for learning abilities? A review of about a dozen such studies concludes that genetic correlations between *g* and learning abilities are substantial but somewhat lower than the genetic correlations among learning abilities (Plomin & Kovas, 2005). This result suggests that most (but not all) generalist genes that affect learning abilities are even more general in that they also affect other sorts of cognitive abilities included in the *g* factor.

## SPECIALIST GENES AND SPECIALIST ENVIRONMENTS

As we have shown, genetic correlations among learning abilities and disabilities are substantial—about .70 on average—which suggests that what they have in common is largely genetic in origin. However, genetic correlations are less than 1.0, which means that genes also contribute to making children better at some abilities than others. In other words, some relatively specialist genes (influencing some abilities but not others) also exist. As mentioned earlier, when DNA research identifies QTLs responsible for genetic influence on reading ability, we predict that most of the QTLs will also be associated with mathematics ability. However, we also predict that some of these QTLs will not be associated with mathematics. Because genetic influence on learning abilities is substantial, such specialist genes contribute importantly to dissociations among learning abilities and disabilities even though most genes are generalists.

Multivariate genetic research also has an interesting story to tell about environmental influences on learning abilities and disabilities. Genetic research distinguishes two types of environmental influences. Those that make family members similar are called *shared environment*. The rest, those that do not contribute to resemblance among family members, are called *nonshared environment*, and this category also includes error of measurement. Multivariate genetic analyses indicate that shared environmental influences are generalists: Shared environmental correlations among learning and cognitive abilities are as high as genetic correlations. For example, in the two recent studies, the shared-environmental correlation was .74 between reading and mathematics at 7 years (Kovas, Harlaar, Petrill, & Plomin, 2005), and the average shared-environmental correlation was .86 between five components of mathematics at 10 years (Kovas et al., 2007). An obvious hypothesis that has not yet been rigorously tested is that some monolithic factors such as the family's socioeconomic status or school quality might be responsible for these generalist shared-environmental effects.

In contrast to these generalist effects of shared environment, nonshared environmental effects are specialists: Nonshared environmental correlations are low. For example, in the same two studies, the nonshared environmental correlation was .39 between reading and mathematics at 7 years (Kovas et al., 2005), and the average nonshared environmental correlation was .24 between five components of mathematics at 10 years (Kovas et al., 2007).

Nearly all research attempting to identify specific sources of nonshared environment has focused on family environments rather than school environments and on personality and behavior problems rather than learning abilities. Nonetheless, such research should be informative for future research that will attempt to identify nonshared environments that affect learning abilities. A meta-analysis of 43 papers relating differential family experience of siblings to differential outcomes concluded that “measured nonshared environmental variables do not account for a substantial portion of nonshared variability” (Turkheimer & Waldron, 2000, p. 78).

The search for nonshared environments might best begin outside the family. For example, initial research supports the hypothesis that peer influence may be an important candidate for a nonshared environment as siblings make their own individual ways in the world outside their family (Iervolino et al., 2002). However, peers would not seem to be a likely explanation for why nonshared environmental factors change so much from year to year, nor why nonshared environmental factors differ from one academic subject to another (Kovas, Haworth, Dale, & Plomin, in press). Perceptions of the environment may be an important direction for research because they are specific to the child. A recent study of 3,000 pairs of 9-year-old twin pairs found that children's perceptions of school experiences were significantly but modestly influenced by genetic factors (20% of the variance), but that most of the variance (65%) was due to nonshared environment (Walker & Plomin, 2006). However, the problem is that these nonshared environmental experiences hardly relate to nonshared environmental variance in academic achievement.

We also need to consider the possibility that chance contributes to nonshared environment in terms of random noise, idiosyncratic experiences, or the subtle interplay of a concatenation of events. However, chance might only be a label for our current ignorance about the environmental processes by which children—even pairs of MZ twins—in the same family and same classroom come to be so different.

Even though we have a long way to go to understand the nonshared environmental influences that are the source of specialist environments, there are important implications now of thinking about specialist environments in relation to education. Almost all work on school environments focuses on shared environmental factors such as family background and school and teacher quality. However, such shared environmental influences have modest effects and, at least for cognitive abilities, decline sharply in importance from childhood to adolescence (Deary et al., 2006). Moreover, shared environmental influences act as generalists. More important, and of increasing importance during development, are nonshared environmental influences. As we have described, multivariate genetic research shows that these environmental factors primarily work as specialists contributing to differences in children's performances in different areas. One implication is that educational influences might have their greatest impact on remediating discrepant performances among learning abilities (such as differences in reading and mathematics) and discrepancies between learning abilities and cognitive abilities, which is one way to view the topic of over- and under-achievement.

## IMPLICATIONS OF GENERALIST GENES

Definitive proof of the importance of generalist genes will come from molecular-genetic research that identifies DNA associated with learning and cognitive abilities and disabilities. The multivariate genetic research reviewed here leads to a clear prediction: Most (but not all) genes found to be associated with a particular learning ability or disability will also be associated with other learning abilities and disabilities. In addition, most (but not all) of these generalist genes for learning abilities (such as reading and mathematics) will also be associated with other cognitive abilities (such as memory and spatial ability).

A major reason why identification of genes has been slower than anticipated is that there are likely to be many more genes (QTLs) with much smaller effect sizes than had been anticipated, which means that larger studies with greater power to detect small effects are needed (Zondervan & Cardon, 2004). Optimism is warranted with the advent of completely new approaches such as whole-genome association studies involving thousands of DNA markers genotyped on microarrays (slides the size of a postage stamp that contain millions of DNA sequences to which single stranded DNA or RNA can hybridise; Carlson, Eberle, Kruglyak, & Nickerson, 2004), including microarray genotyping of DNA pooled across large samples of learning-disabled individuals and controls (Butcher, Kennedy, & Plomin, 2006). The good news from the generalist-genes theory is the prediction that the same set of genes is associated with most learning disabilities. Studies that collect data on multiple phenotypes can empirically test the generalist-genes hypothesis by testing whether genes found to be associated with one phenotype (e.g., reading) also relate to other phenotypes in the same sample.

Although no genes have as yet been reliably identified as associated with learning disabilities, several linkages to chromosomal regions have been found for learning disabilities. These QTL linkage results provide some support for the theory of generalist genes. For example, for reading disability the linkages are general. That is, the same linkages appear across measures of diverse reading processes, including orthographic coding, phonological decoding, word recognition, and rapid naming (e.g., Fisher & DeFries, 2002).

When the generalist genes are identified, they will greatly accelerate research on general mechanisms at all levels of analysis from genes to brain to behavior. We have recently discussed implications of generalist genes for cognitive and brain sciences (Kovas & Plomin, 2006). Implications of generalist genes for translational research are also far-reaching. Multivariate genetic research reviewed in this article suggests that genetic “diagnoses” of learning disabilities differ from traditional diagnoses: From a genetic perspective, learning disabilities are not distinct diagnostic entities. The same set of generalist genes affects learning abilities and disabilities. Discrepancies in children's profiles of performance are largely due to specialist environments.

